# ICONIC: An R Package for Integrating Instrumental Variable- and Negative-Control-Informed Causal Discovery and Diagnostics in Multiomic Studies

**DOI:** 10.64898/2026.08.26.26361466

**Published:** 2026-08-31

**Authors:** Sean T. Bresnahan, Charis Xiong, Taylor Head, Yung-Han Chang, Arjun Bhattacharya, Jonathan Y. Huang

## Abstract

Unmeasured confounding threatens causal inference and replicability in observational multi-omic studies across variable environments. Genetic instrumental variables (Mendelian randomization) and negative-control calibration each address complementary sources of unmeasured confounding, yet no existing framework unifies them for omics-scale mediation analysis. We introduce ICONIC, an R package that embeds genetic instruments and negative controls within a proximal causal inference framework for total-effect and mediation analysis. ICONIC implements eight estimators spanning five confounding-control strategies, supports continuous, binary, and time-to-event outcomes, and provides extensive diagnostics including sensitivity analyses that map estimator performance across plausible assumptions. Ground-truth benchmarks are calibrated to real-omics covariance structures via a hybrid generative model (GAN + feature-level Gaussian copula) rather than parametric simulation, and a companion planning tool predicts performance gains from collecting additional omic data. We demonstrate ICONIC in two case studies: screening for placental transcriptomic mediators of gestational diabetes on birth weight (n = 164), and tumor-expression mediators of smoking intensity on lung cancer survival (n = 494). Notably, ICONIC’s diagnostics recommended different estimation strategies across the two scenarios, reflecting differences in the likely influence of unmeasured confounding. ICONIC is freely available at https://github.com/sbresnahan/iconic/ .

## Full Text Availability

The license terms selected by the author(s) for this preprint version do not permit archiving in PMC. The full text is available from the preprint server.

